# Discovery of a Novel Cyclopeptide as Tyrosinase Inhibitor for Skin Lightening

**DOI:** 10.1111/srt.70207

**Published:** 2025-09-12

**Authors:** Huailong Chang, Kan Tao, Hu Huang, Jinping Jia, Shah Nawaz Khan, Jiahua Cui

**Affiliations:** ^1^ Global R&D Center Shanghai Chicmax Cosmetic Co. Ltd Shanghai China; ^2^ School of Chemistry and Chemical Engineering Shanghai Jiaotong University Shanghai China; ^3^ Department of Pharmacy University of Malakand Khyber Pakhtunkhwa Pakistan

**Keywords:** chemical synthesis, cosmetics, cyclopeptide, molecular docking, skin lightening, tyrosinase inhibitors

## Abstract

**Background:**

Melanin synthesis plays a crucial role in skin pigmentation, and inhibiting tyrosinase, the key enzyme in melanin production, is a primary strategy for developing skin‐lightening agents. This study investigates the tyrosinase inhibitory potential of **CHP‐9**, a novel cyclopeptide, and evaluates its cytotoxicity and efficacy as a cosmetic depigmenting agent.

**Methods:**

**CHP‐9** was synthesized via a solid‐phase peptide synthesis strategy. The tyrosinase inhibitory activity was assessed using an enzymatic assay, while its effects on melanin content were evaluated in cultured human melanocytes. The MTT assay was performed to assess cytotoxicity across a range of **CHP‐9** concentrations (0.0781–10 mg/mL). Molecular docking simulations were conducted to elucidate the interaction between **CHP‐9** and human tyrosinase (PDB ID: 5M8M). Statistical analysis was performed using GraphPad Prism Software, and significance was determined via one‐way ANOVA.

**Results:**

**CHP‐9** exhibited significant tyrosinase inhibition (28.57% at 1% concentration) and reduced melanin content in treated melanocytes from 30.90 ± 1.13 to 23.51 ± 1.14 µg/mL. Cytotoxicity assays confirmed CHP‐9's high biocompatibility, with cell viability exceeding 90% at concentrations up to 2.5 mg/mL. Docking studies revealed strong binding affinity between **CHP‐9** and key tyrosinase residues via hydrogen bonding, supporting its inhibitory mechanism.

**Conclusions:**

**CHP‐9** exhibited significant tyrosinase inhibition (28.57% at 1% concentration) and reduced melanin content in melanocytes, while maintaining over 90% cell viability at effective doses. These findings suggest that CHP‐9 is a safe and effective candidate for cosmetic skin‐lightening applications. Further research is needed to enhance formulation stability and evaluate long‐term efficacy in vivo.

## 1 Introduction

Hyperpigmentation is a prevalent cosmetic concern characterized by excessive melanin deposition in sun‐exposed skin regions due to heightened melanogenesis [1]. While naturally sun‐induced melanin production offers photoprotective benefits and contributes to a natural tan, localized overaccumulation of melanin often results in undesirable dark spots, posing significant cosmetic challenges [2, 3].

Tyrosinase is a key enzyme in the polyphenol oxidase family and serves as the central catalyst in melanin biosynthesis [4, 5, 6, 7] by converting tyrosine into L‐DOPA and subsequently oxidizing L‐DOPA to O‐quinone, which undergoes spontaneous reactions to synthesize melanin [8, 9]. Overproduction of melanin driven by tyrosinase hyperactivity is directly linked to hyperpigmentation disorders such as solar lentigo, chloasma, and freckles, which can markedly alter phenotypic appearance [10]. Beyond its role in skin pigmentation, dysregulated tyrosinase activity plays a crucial role in neurodegenerative conditions like Parkinson's and Alzheimer's diseases [11, 12] and food science [13, 14]. Given these implications, targeted inhibition of tyrosinase's catalytic activity emerges as a promising strategy for mitigating hyperpigmentation (e.g., skin lightening) and addressing other conditions as well.

The readily available ascorbic acid [15, 16], kojic acid [17], arbutin [18, 19], and hydroquinone [20, 21, 22] are among the myriad of tyrosinase inhibitors. Nonetheless, the drawbacks of these inhibitors make them blemished due to the potential carcinogenicity, high cellular toxicity, instability, and off‐odor propensities [22, 23, 24]. With the growing consumer awareness toward the possible side effects of these inhibitors, there is a high demand for naturally occurring tyrosinase inhibitors as a safer alternative.

Bioactive peptides derived from food have emerged as promising alternatives due to their potential effectiveness, availability, targeting capabilities, safety, and specificity [25, 26]. However, despite the potential theoretical and practical application of these peptides, they always encounter challenges, along with their stability and selectivity, which remain a critical concern for peptide tyrosinase inhibitors [25, 27, 28]. These limitations are evident in both in vivo and in vitro environments, where many peptide compounds undergo rapid deactivation. This instability reduces their ability to provide sustained effects, ultimately compromising therapeutic efficacy and the overall patient experience. Furthermore, the bioavailability of these inhibitors presents another challenge. Traversing cell membrane is impeded due to the large molecular weight of peptide compounds, leading to restricted drug concentration in the body to reach minimum therapeutic levels [29, 30]. Addressing this issue involves enhancing drug design; for instance, the drug bioavailability could be improved by incorporating suitable carriers or modifying groups. These challenges significantly limit the practical application of peptide tyrosinase inhibitors in clinical settings.

## 2 Methods Introduction

### 2.1 Materials

Methyl thiazolyl tetrazolium (MTT) was procured from Sigma‐Aldrich. DMEM/F12 Medium, fetal bovine serum (FBS), and penicillin/streptomycin were obtained from Gibco. Phosphate‐buffered saline (PBS) was sourced from Solarbio, China. Analytical Grade Melanin, tranexamic acid, and L‐DOPA were purchased from Sigma. Glabridin and kojic acid were acquired from Olibio Co. Ltd. and Yuanye Biotechnology Co. Ltd., respectively. All other reagents were obtained from commercial sources and used as received without further purification.

### 2.2 Experiment

**SCHEME 1:**
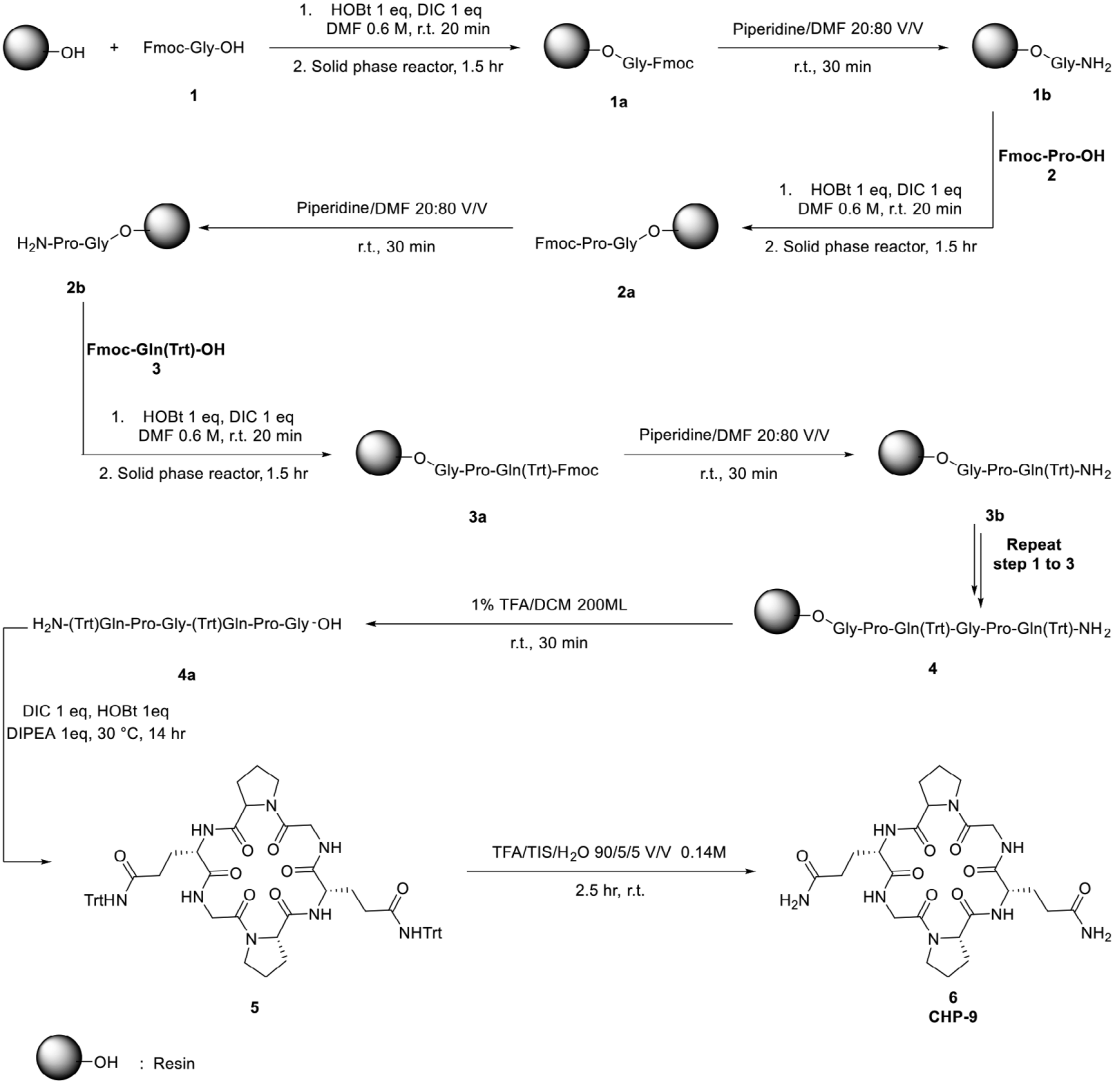
Synthetic strategy of cyclopeptide (**CHP‐9**).

CHP‐9 was synthesized using solid‐phase peptide synthesis (SPPS), Scheme 1. The peptide sequence was assembled from Fmoc‐protected amino acids (Fmoc‐Gly‐OH, Fmoc‐Pro‐OH, Fmoc‐Gln(Trt)‐OH) on a solid‐phase resin reactor flask. After linear peptide assembly, the sequence was cyclized, deprotected, and purified via preparative reverse‐phase C18 chromatography using methanol and water followed by lyophilization to obtain the pure compound (cyclopeptide **CHP‐9**) Cyclo(Gln‐Gly‐Pro‐Gln‐Gly‐Pro) (**6**), with a final purity of 99.2%, confirmed by HPLC analysis. HRMS (ESI‐TOF): m/z [M + Na]^+^ calcd for C_24_H_36_O_8_N_8_Na, 587.2554; found, 587.2549.

### 2.3 Cell Culture and Cytotoxicity Evaluations

Human melanocytes (Batch number: MC210817; Guangdong Biocell Biotechnology Co. Ltd.) were cultured in DMEM/F12 medium supplemented with 20% FBS (Hyclone) and 100 U/mL penicillin at 37°C with 5% CO_2_ atmosphere.

For cytotoxicity, evaluation of peptides on human melanocyte cells was assessed using the standard MTT assay [31]. Cells were seeded in 96‐well plates at a density of 10 000 cells per well and incubated for 12 h under standard culture conditions. After adherence, cells were treated with eight gradient concentrations of target peptides ranging from 0.0781 to 10 mg/mL or a positive control (nonapeptide‐1 and tranexamic acid alone or in combination 0.3% v/v) for 24 h. A negative control group (culture medium containing 10% Dimethyl sulfoxide (DMSO), v/v) was included. Post‐treatment, 0.5 mg/mL MTT reagent was added to each well, followed by 4 h of incubation at 37°C. Formazan crystals formed in viable cells were dissolved in 150 µL DMSO, and absorbance was measured at 490 nm using a Synergy multimode reader (BioTek, USA). Each concentration was tested in triplicate. Statistical significance was determined via one‐way ANOVA (GraphPad Prism 5). All experiments were conducted in triplicate (*n* = 3).

### 2.4 Determination of Melanin Content in Melanocyte

Upon reaching 80%–90% confluence, human melanocyte cells were dissolved with 0.25% trypsin to create a single‐cell suspension. Then, the cells were seeded in 6‐well plates at 200 000 cells per well and cultured for 12 h in a 37°C humidified incubator (5% CO_2_, V/V). When the confluence of cells reached 40%–60%, they were incubated with specific peptides or the positive control for 24 h. Following the incubation period, the supernatant was removed and the adherent cells were washed with PBS before being dispersed using trypsin (700 µL/well, 0.25%). The dispersed cells were collected and centrifuged at 10 000 rpm for 10 min. The cell pellet from each well was then treated with a mixture of distilled water (200 µL), anhydrous ethanol (500 µL), and ether (500 µL). The mixture was vortexed and allowed to stand at room temperature for 20 min, followed by centrifugation at 3000 rpm for 5 min. The precipitate was dissolved in 1 mL of 1 M NaOH containing 10% DMSO, then incubated at 80°C in a water bath for 40 minutes. After incubation, the solution was transferred to a 96‐well culture plate for optical density measurement at 405 nm. Each analysis was performed in triplicate and results were expressed as mean ± standard deviation (SD).

### 2.5 Tyrosinase Inhibition Assay

The inhibition of tyrosinase activity was assessed according to the reported method [32]. The 6‐well plates were incubated at 37°C with human melanocyte cells at a density of 2.5×10^5^ cells per well in a humidified atmosphere (5% CO_2_) for 24 h. Subsequently, these cells were exposed to glabridin (0.00625%, V/V) as the positive control, along with other tested compounds. Following treatment, cells were incubated at 37°C for 24 h using identical conditions. After treatment, cells in each well were rinsed with a PBS buffer solution (500 µL) and lysed with a lysate buffer (100 µL containing 0.5% sodium deoxycholate) at approximately 0°C for 1 h. The resulting cell lysate was then centrifuged at 12 000 rpm at 4°C for 20 min. The protein concentration in the supernatant was quantified by a BCA Assay Kit (Beyotime Biotechnology, China). Samples were transferred to a 96‐well plate in a total volume of 100 µL (90 µL of lysate supernatant and 20 µL of 0.1% L‐DOPA) and incubated for 60 min at 37°C. The absorbance was measured at 475 nm using a Synergy multimode reader (BioTek Corporation, USA).

### 2.6 Molecular Docking

The cyclopeptide **CHP‐9**, a potent inhibitor in tyrosinase inhibition assay, was docked into the active site cavity of human tyrosinase (PDB ID: 5M8M) using the MOE 2008 docking program after removing all water molecules from the crystal structure before the experiment to ensure precise docking results. The Protonate 3D application was added with hydrogens and partial charges. The docking site was defined for residues within an 8 Å radius of kojic acid. The initial 3D conformation of **CHP‐9** was optimized in ChemBio3D Ultra using the MM2 energy minimization method. Default parameter values were applied for docking, with only the scoring function modified to ASE Scoring from the typical London dG. This scoring adjustment identified the best pose.

To assess the feasibility of the docking program for the ligand binding to human tyrosinase, we initially selected tyrosinase‐related protein 1 in complex with kojic acid (PDB ID: 5M8M) and the docking structure of kojic acid was comparable to its crystallographic structure. This result implied that the MOE docking program is appropriate for identifying a binding model between CHP‐9 and human tyrosinase.

## 3 Results and Discussion

### 3.1 Cytotoxicity Evaluation

The cytotoxicity of cyclopeptide **CHP‐9** was assessed to determine its safety for cosmetic applications, along with tranexamic acid alone, tranexamic acid plus nonapeptide‐1, and tranexamic acid combined with nonap3eptide‐1 and cyclopeptide CHP‐9, against human melanocyte cells.

**FIGURE 1:**
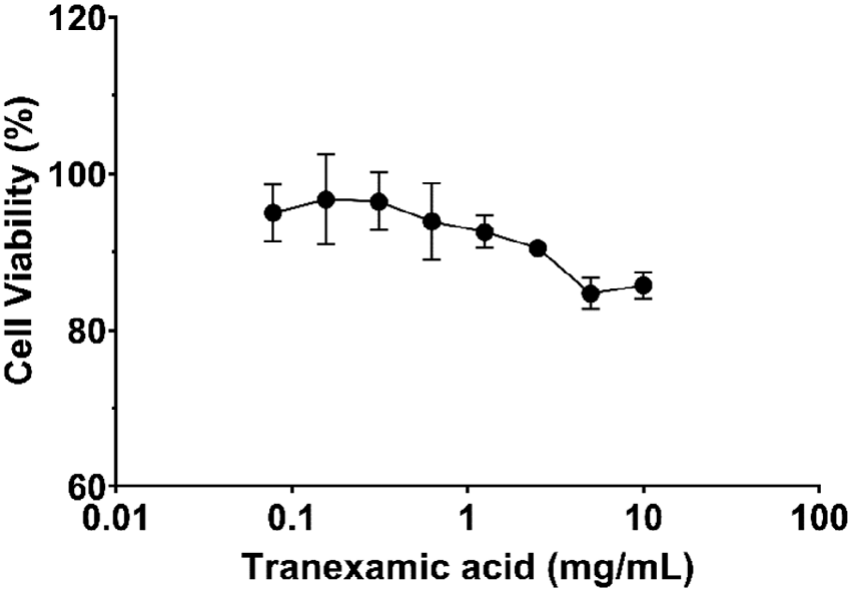
Growth inhibition of tranexamic acid against human melanocyte cells.

As illustrated in Figure 1, tranexamic acid exhibited minimal cytotoxicity in human melanocytes at concentrations up to 2.5 mg/mL, with cell viability remaining above 90% (Scheme 1). A slight reduction in cell viability was observed at higher concentrations (5 and 10 mg/mL), but these effects were not statistically significant.

**FIGURE 2:**
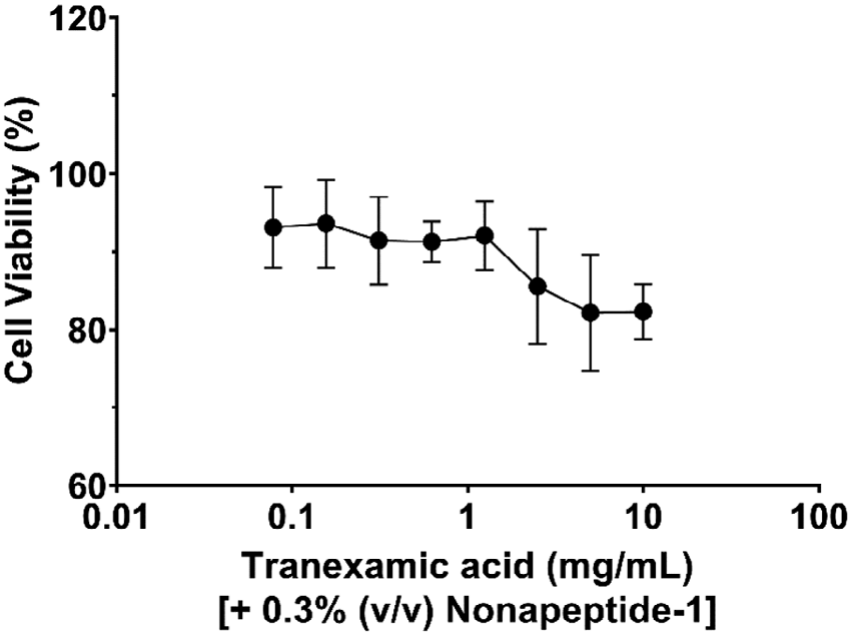
Growth inhibition of tranexamic acid plus nonapeptide‐1 (0.3%, v/v) against human melanocyte cells.

Additionally, incubating cells with tranexamic acid and nonapeptide‐1 at 0.3% (v/v) did not significantly affect cell viability (Figure 2). A slight decrease in cell viability was observed when the concentration of tranexamic acid was increased to 5 mg/mL, but overall, no significant cytotoxic effects were detected.

**FIGURE 3:**
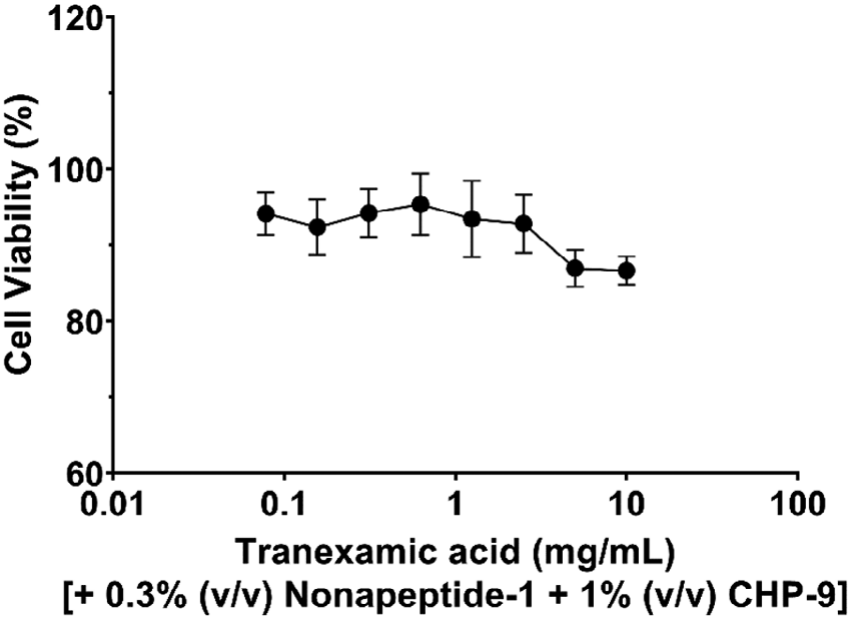
Growth inhibition of tranexamic acid (1 mg/mL), nonapeptide‐1 (0.3%, V/V), and cyclopeptide (CHP‐9) against human melanocyte cells.

For the group in which human melanocyte cells were treated with tranexamic acid (1 mg/mL), nonapeptide‐1 (0.3%, v/v), and cyclopeptide **CHP‐9**, cell viability was evaluated using the MTT assay. As shown in Figure 3, even with **CHP‐9** at a concentration of 2.5 mg/mL, the mixture did not significantly affect cell growth, confirming that **CHP‐9** does not introduce additional cytotoxicity to melanocytes.

These findings suggest that **CHP‐9** is a biocompatible tyrosinase inhibitor (noncytotoxic to human melanocytes at tested concentrations), unlike traditional skin‐lightening agents such as hydroquinone, which exhibit cytotoxic effects at similar concentrations. These results support the safe application of **CHP‐9** in cosmetic formulations.

CHP‐9's low cytotoxicity may be attributed to its structural properties as a cyclic peptide. Cyclic peptides often demonstrate enhanced stability and selective bioactivity compared to linear counterparts, allowing them to interact specifically with target proteins (e.g., tyrosinase) while sparing nontarget cellular components. The lack of membrane‐disruptive domains and the relatively small, hydrophilic nature of CHP‐9 may limit off‐target effects and cellular stress responses. Furthermore, its moderate binding affinity, as revealed through docking, suggests a balanced interaction that avoids nonspecific enzyme or receptor activation, thus minimizing cytotoxic outcomes.

The cytotoxicity assay results are critically relevant to assessing the practical potential of CHP‐9 as a tyrosinase inhibitor. Many conventional tyrosinase inhibitors, such as hydroquinone, have shown efficacy but suffer from high cytotoxicity, limiting their safe use in cosmetic formulations. In contrast, CHP‐9 maintained high cell viability (>90%) across effective concentrations, indicating that its melanin‐reducing and enzyme‐inhibiting effects occur without compromising cell health. This suggests that CHP‐9 can provide therapeutic efficacy with a favorable safety margin, making it a strong candidate for long‐term application in skin‐lightening products.

### 3.2 Effect of Cyclopeptide on Intracellular Tyrosinase Activity and Melanin Content in Melanocyte Cells

**FIGURE 4:**
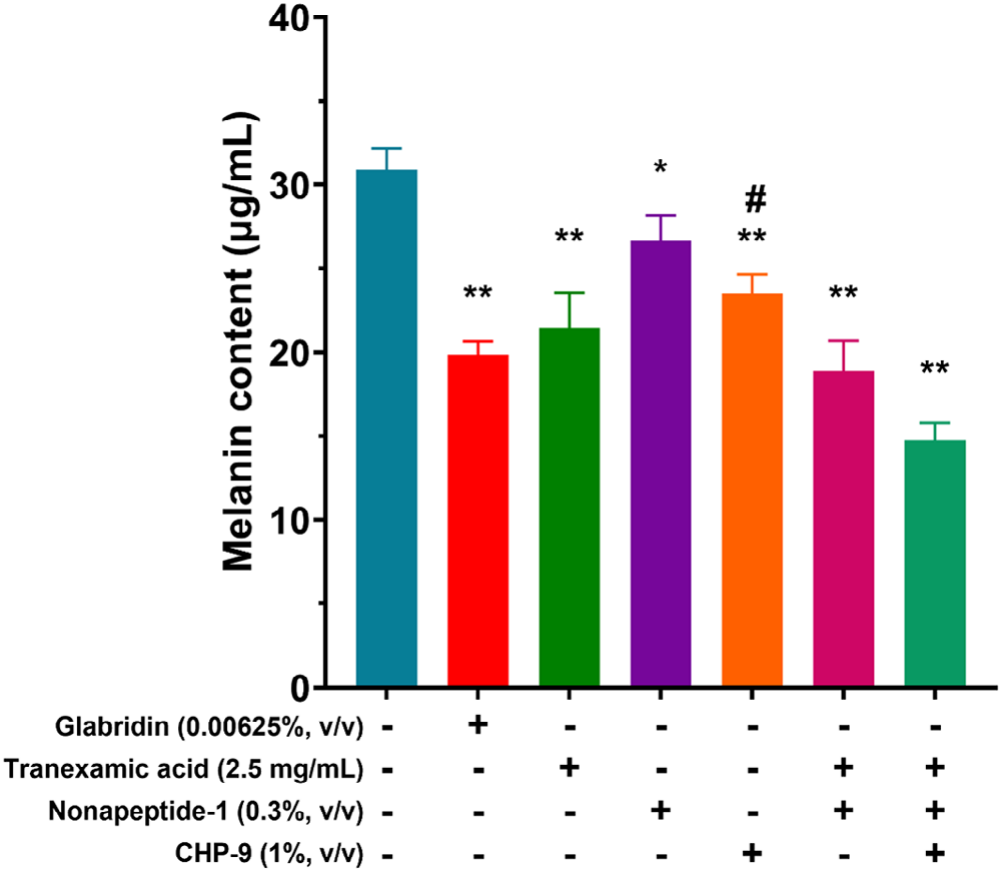
Inhibition of melanin production by cyclopeptide and related compounds (data shown are mean ± SD analyzed by one‐way ANOVA with multiple comparison testing for the vehicle group and the indicated corresponding inhibition group. *n* = 3. *p < 0.05 and **p < 0.01 for comparison between the vehicle group and the indicated group; #*p* < 0.05 for comparison between nonapeptide‐1 and the indicated CHP‐9 group).

The melanin‐reducing effects of **CHP‐9** and related compounds were investigated, as shown in Figure 4. The bar graph illustrates melanin content in human melanocyte cells following treatment with various compounds. The untreated control group displayed the highest melanin content (∼30.90 µg/mL), which served as a baseline.

Treatment of human melanocytes with **CHP‐9** (1%) alone significantly reduced melanin levels to ∼23.51 µg/mL, demonstrating its ability to inhibit melanin production effectively.

Tranexamic acid (2.5 mg/mL) and nonapeptide‐1 (0.3%, v/v) also exhibited melanin‐reducing effects, though not as strongly as **CHP‐9** alone. The combination of tranexamic acid and nonapeptide‐1 resulted in a further decrease in melanin levels compared to their individual effects, suggesting a potential synergistic effect.

Additionally, glabridin (0.00625%, v/v), a well‐known skin‐lightening agent, was included in the study. It showed a strong inhibitory effect on melanin production, reinforcing its established role in tyrosinase inhibition.

Notably, the combination of tranexamic acid, nonapeptide‐1, and **CHP‐9** resulted in a significant reduction in melanin content, comparable to or even greater than the effects of individual compounds. This suggests that **CHP‐9** enhances the skin‐lightening potential of tranexamic acid and nonapeptide‐1, making it a promising candidate for cosmetic applications targeting hyperpigmentation.

Figure 4 demonstrates that **CHP‐9** effectively reduces melanin production, both alone and in combination with other compounds. Its inhibitory effect is comparable to glabridin, a well‐established skin‐lightening agent. Overall, these findings suggest that CHP‐9 is a safe and effective ingredient for cosmetic applications aimed at reducing hyperpigmentation.

The inhibitory effects of CHP‐9 on melanin content may be attributed to its ability to bind specifically to the active site of tyrosinase, blocking its catalytic activity. This disruption in tyrosinase function prevents the conversion of L‐DOPA to dopachrome, ultimately reducing melanin biosynthesis in melanocytes. Additionally, the stable structure of CHP‐9 allows for sustained interaction with tyrosinase, leading to prolonged inhibitory effects. Its selective binding may also minimize interference with other intracellular signaling pathways, thereby reducing toxicity while effectively inhibiting melanin formation.

### 3.3 Tyrosinase Inhibitory Activity Evaluation

**FIGURE 5:**
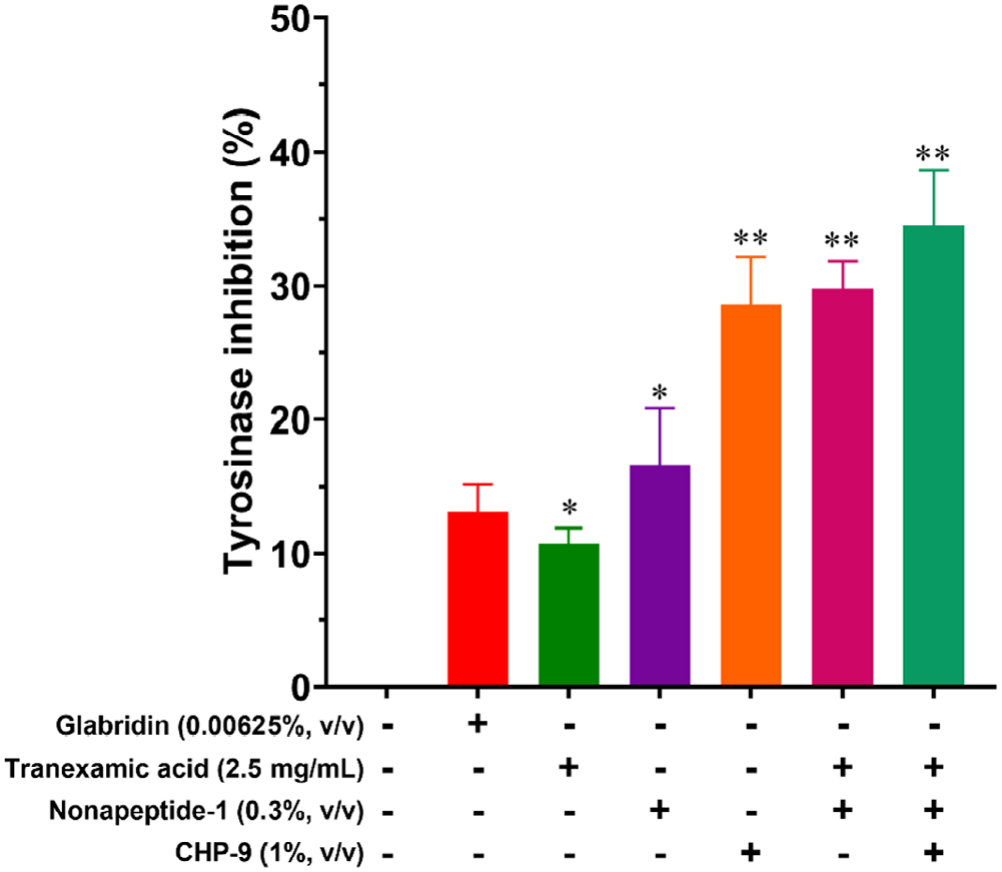
Tyrosinase inhibitory activity of synthetic peptides (data shown are mean ± SD analyzed by one‐way ANOVA with multiple comparison testing for the vehicle group and tested groups. *n* = 3. *p < 0.05 and **p < 0.01 for comparison between the vehicle group and the indicated group).

The tyrosinase inhibitory activities of the tested compounds were evaluated according to previously reported methods [25] and the results are presented in Figure 5. Glabridin, a well‐known tyrosinase inhibitor [33], was used as the positive control, demonstrating an inhibition rate of approximately 14% at a concentration of 0.00625% (v/v). Among the tested compounds, the cyclopeptide **CHP‐9** (at 1% concentration) exhibited the highest tyrosinase inhibition, with a rate of 28.57%, which is about twice as potent as glabridin.

The inhibition rates of tranexamic acid (2.5 mg/mL) and nonapeptide‐1 (0.3% v/v) were measured at 10.71% and 16.67%, respectively, suggesting moderate tyrosinase inhibitory activity for both compounds. Notably, the combination of tranexamic acid and nonapeptide‐1 showed a synergistic effect, achieving an inhibition rate of 29.76%, which is slightly higher than the sum of their individual inhibition rates (27.38%).

The highest inhibition rate (34.52%) was observed when **CHP‐9** (1%) was combined with tranexamic acid (2.5 mg/mL) and nonapeptide‐1 (0.3% v/v), demonstrating the most potent inhibitory efficacy among all tested groups.

The statistical significance of the results was analyzed using one‐way ANOVA with multiple comparison testing. The differences in inhibition rates between the vehicle control and tested groups were statistically significant (*p* < 0.05, *p* < 0.01), as indicated in Figure 5. The observed differences suggest that **CHP‐9**, both alone and in combination with other compounds, effectively inhibits tyrosinase activity, further supporting its potential application in cosmetic formulations.

These results suggest that **CHP‐9** is a highly effective tyrosinase inhibitor, exhibiting significantly stronger activity compared to the standard positive control, glabridin. The synergistic enhancement observed in the tranexamic acid and nonapeptide‐1 combination indicates that their combined effect is greater than the sum of their individual inhibition rates, likely due to complementary mechanisms of tyrosinase inhibition.

Moreover, the tri‐combination of **CHP‐9**, tranexamic acid, and nonapeptide‐1 displayed the most potent inhibitory activity, suggesting that multitarget approaches may be more effective in tyrosinase inhibition. This combination may exert its effects through different inhibition pathways, leading to a higher overall suppression of tyrosinase activity.

These findings highlight the potential application of **CHP‐9**, alone or in combination with other agents, in skin‐lightening and hyperpigmentation treatments. Further studies will be necessary to elucidate the precise mechanism underlying these synergistic effects and to assess their in vivo efficacy.

### 3.4 Molecular Docking Between the Cyclopeptide and Tyrosinase

Recently, computer‐based simulation, known as molecular docking, has become widely used to investigate potential interaction mechanisms between inhibitors and corresponding targets. To gain insights into the molecular determinants modulating the inhibitory activity of the cyclopeptide **CHP‐9** against tyrosinase, we perform molecular docking studies of **CHP‐9** with human tyrosinase using the MOE 2008 software based on the x‐ray crystal structure of human tyrosinase‐related protein 1 in complex with kojic acid in the active site cavity (PDB ID: 5M8M).

**FIGURE 6:**
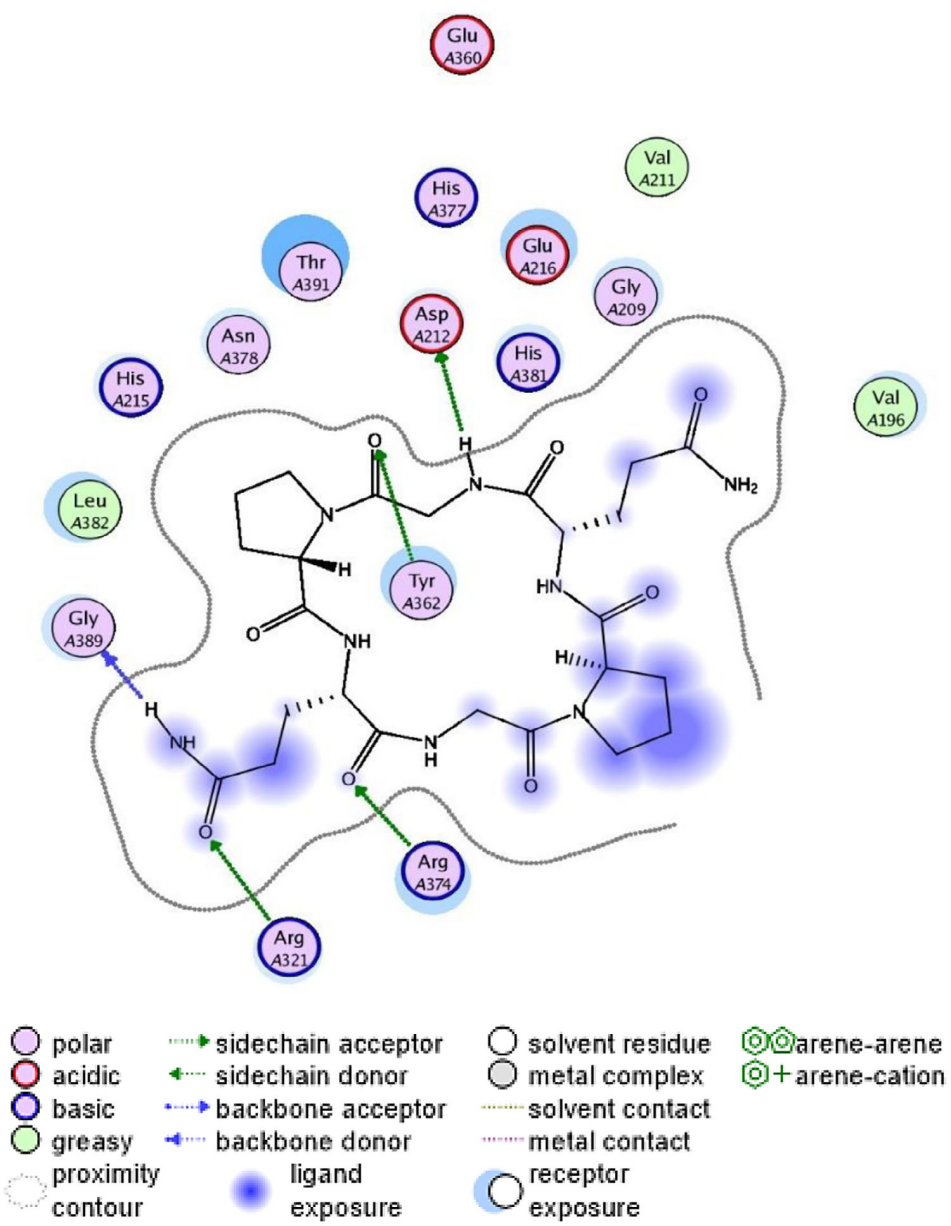
The molecular docking simulation results of the cyclopeptide **CHP‐9** at the active site of tyrosinase (2D‐interactions).

As shown in Figure 6, the cyclopeptide **CHP‐9** is bound within the binding cavity, positioned above the surface of the catalytic center. Different interaction types can significantly influence the structure binding affinity and stability between the tyrosinase and **CHP‐9** peptide. Specifically, a total of six hydrogen bonds were formed (one cation hydrogen bond and five conventional hydrogen bonds) between peptide **CHP‐9** and tyrosinase, including six amino acid residues in tyrosinase, that is, Asp212, Tyr362, His321, Gly389, Arg374, and His382.

Additionally, the hydrophobic aliphatic residue leucine382 plays a pivotal role in tyrosinase inhibition by directly interacting with tyrosinase to block dopaquinone formation. Considering the results from molecular docking, cyclopeptide **CHP‐9** acts as a tyrosinase inhibitory peptide, and we observed that hydrogen bonding was crucial in influencing the binding affinities and structure stabilities of the peptide–tyrosinase complexes, thereby blocking the tyrosinase activity.

## 4 Conclusion

Melanin content is a key determinant of skin pigmentation, and inhibiting melanin formation is a crucial strategy for achieving skin‐lightening effects. In this study, we identified CHP‐9, a novel cyclopeptide, as a potent and safe human tyrosinase inhibitor. CHP‐9 demonstrated significant tyrosinase inhibition, with an inhibition rate of 28.57% at 1% concentration. Furthermore, in the melanin formation assay, exposure to **CHP‐9** alone led to a reduction in cellular melanin content from 30.90 ± 1.13 to 23.51 ± 1.14 µg/mL, indicating its ability to effectively suppress melanin biosynthesis. Molecular docking studies further confirmed the strong binding affinity of **CHP‐9** to tyrosinase, primarily facilitated by hydrogen bonding interactions.

Despite these promising results, some limitations and challenges remain. Although **CHP‐9** has shown strong in vitro tyrosinase inhibitory activity, further in vivo studies are required to evaluate its efficacy, bioavailability, and long‐term safety in skin applications. Additionally, optimizing the formulation and delivery system of **CHP‐9** in cosmetic products is essential to enhance its stability and penetration into the skin. Future research should also explore structure–activity relationship (SAR) studies to improve its inhibitory potency and selectivity.

Overall, these findings suggest that **CHP‐9** holds great potential as a bioactive compound for skin‐lightening applications in cosmetics. However, further investigation is necessary to overcome existing challenges and fully realize its therapeutic and commercial potential.

## Ethics Statement

Ethical review and approval were not required for this study as it did not involve humans or animals.

## Conflicts of Interest

The authors declare no conflicts of interest.

## Supporting information




**Scheme 1**: Deprotection of Glycine
**Scheme 2**: deprotection of Proline
**Scheme 3**: Synthesis of linear peptide:
**Scheme 4**: cleavage of the linear peptide from resin.
**Scheme 5**: Cyclization of linear peptide:
**Scheme 6**: Deprotection of cyclized peptide
**Table S1**: 1H‐NMR Data (D2O, 600 MHz) of Compound CHP‐9 (δ in ppm and J in Hz)
**Table SII**: 13C‐NMR Data (D2O, 600 MHz) of Compound CHP‐9 (δ in ppm and J in Hz)
See Supplementary Material (Table S1 and Table S2) for detailed NMR data of compound CHP‐9.

## Data Availability

The synthesis and characterization data supporting the findings of this study are provided in the Supporting Information, along with a preprint publicly accessible at [DOI: 10.20944/preprints202410.1006.v1]. Any additional data required will be available from the corresponding authors upon reasonable request.
