# Supplementary material for: Discovery of a Novel Cyclopeptide as Tyrosinase Inhibitor for Skin Lightening
Source: Skin Res Technol. 2025 Sep 12;31(9):e70207. doi: 10.1111/srt.70207 (PMC12430830; doi:10.1111/srt.70207)
Supplement: Supplementary file 1 — Scheme 1: Deprotection of Glycine Scheme 2: deprotection of Proline Scheme 3: Synthesis of linear peptide: Scheme 4: cleavage of the linear peptide from resin. Scheme 5: Cyclization of linear peptide: Scheme 6: Deprotection of cyclized peptide Table S1: 1H‐NMR Data (D2O, 600 MHz) of Compound CHP‐9 (δ in ppm and J in Hz) Table SII: 13C‐NMR Data (D2O, 600 MHz) of Compound CHP‐9 (δ in ppm and J in Hz) See Supplementary Material (Table S1 and Table S2) for detailed NMR data of compound CHP‐9. [file SRT-31-e70207-s001.docx]

**Supporting Information for**

**Discovery of A Novel Cyclopeptide as Tyrosinase Inhibitor for Skin Lightening**

Huailong Chang^1^, Kan Tao^1^, Hu Huang^1^, Jinping Jia^2^, Shah Nawaz Khan^2,3*^ and Jiahua Cui^2*^

**Affiliations:**

1. *Global R&D Center, Shanghai Chicmax Cosmetic Co., Ltd.*
2. *School of Chemistry and Chemical Engineering, Shanghai Jiaotong University, Shanghai, China*
3. *Department of Pharmacy, University of Malakand, Khyber Pakhtunkhwa, Pakistan*

**1. General Information**

^1^H NMR spectra were recorded on a Varian INOVA-600 spectrometer (600 MHz) with D_2_O as the solvent. Chemical shifts were reported in δ units with TMS as the internal standard. ^13^C NMR spectra were recorded on the Varian INOVA-600 spectrometer (125 MHz). HRMS spectra were measured on an Agilent 1200RPLC-6520 Q-TOF Premier mass spectrometer. The target compound **CHP-9** has been prepared using the reported method (Hexapeptide-9 cyclic peptide and application thereof, CN116284256B).

**2. Synthesis of CycloPeptide CHP-9.**

Cyclopeptide **CHP-9** was synthesized using the following six steps according to the reported procedure ^[1]^.

**Scheme 1:** Deprotection of Glycine

**Step 1:** Into 100 ml three-neck round bottom flask, Fmoc-Gly-OH (4.46 g, 15 mmol) and 1-Hydroxybenzotriazole (HOBt) (2.03 g, 15 mmol) were added at 4 °C, followed by the addition of *N,N*'-Diisopropylcarbodiimide (DIC) (2.3 mL, 15 mmol) dissolved in 25 mL DMF, and stirred for 20 minutes. The reaction mixture was transferred to a 125 mL solid-phase resin reactor flask and stirred for 1.5 hours. Upon completion of the reaction, the mixture was washed with DMF (3 x 65 ml). After washing, 65 ml of Piperidine/DMF (20:80, V/V) solution was added and stirred at room temperature for 30 minutes. The reaction mixture was filtered, washed with DMF (6 x 65 ml), and dried under vacuum to obtain compound **1b** (Scheme 1).

**Scheme 2:** deprotection of Proline

**Step 2:** In a three-neck 100 ml round bottom flask, **1b**, Fmoc-Pro-OH (5.06 g, 15 mmol) and HOBt (2.03g, 15mmol) were added at 4 ℃, followed by the addition of DIC (2.3 mL, 15 mmol) dissolved in 25 mL DMF and stirred for 20 minutes. The reaction mixture was transferred to a 125 mL solid-phase resin reactor flask and stirred for 1.5 hours. Upon completion of the reaction, the mixture was washed with DMF (3 x 65 ml). After washing, 65 ml of Piperidine/DMF (20:80, V/V) solution was added and stirred at room temperature for 30 minutes. The reaction mixture was filtered and washed with DMF (6 x 65 ml), methanol (2 x 65mL), DCM (2 x 65mL) and finally with (2 x 65mL) methanol to deliver compound **2b** (Scheme 2).

**Scheme 3:** Synthesis of linear peptide:

**Step 3:** In a three-neck 100 ml round bottom flask, **2b**, Fmoc-Gln(*Trt*)-OH (9.16 g, 15 mmol) and HOBt (2.03 g, 15 mmol) were added at 4 ℃, followed by the addition of DIC (2.3 mL, 15 mmol) dissolved in 25 mL DMF and stirred for 20 minutes. The reaction mixture was transferred to a 125 mL solid-phase resin reactor flask and stirred for 1.5 hours. Upon completion of the reaction, the mixture was washed DMF (3 x 65 ml). After washing, 65 ml of Piperidine/DMF (20:80, V/V) solution was added and stirred at room temperature for 30 minutes. The reaction mixture was filtered and washed with DMF (6 x 65 ml) to obtain compound **3b**. Then, steps 1 to 3 were repeated, combined and dried under a vacuum to deliver the linear peptide **4** (Scheme 3). The linear peptide proceeded to the next step without further purification.

**Scheme 4:** cleavage of the linear peptide from resin.

**Step 4:** In a 500 ml threeneck round bottom flask, the crude product **4** from step 3 (vacuum dried) was added to 200 mL of 1% TFA/DCM solution and stirred at room temperature for 30 minutes. Upon completion of the reaction, the mixture was filtered and the filtrate was dried to obtain the *Trt*-protected linear peptide (**4b**) H-Gly-Pro-Gln(*Trt*)-Gly-Pro-Gln(*Trt*)-OH (Scheme 4).

**Scheme 5:** Cyclization of linear peptide:

**Step 5:** In a threeneck 2 L round bottom flask, the *Trt*-protected peptide (**4b**) was dissolved in 1.4 L of dichloromethane (DCM), followed by DIC (1.54 mL, 10 mmol), HOBt (1.35 g, 10 mmol), Diisopropyl ethyl amine (DIEA) (1.74 mL, 10 mmol) addition, the reaction mixture was stirred at 30 ℃ for 14 hours to generate Cyclo(Gly)-Pro-Gln(Trt)-Gly-Pro-Gln(Trt)) **5** (Scheme 5). The solvent was removed under reduced pressure and proceeded to the next step without further purification.

**Scheme 6:** Deprotection of cyclized peptide

**Step 6:** In a 150 ml round bottom flask, the deprotection of crude product (**5**) Cyclo(Gly-Pro-Gln(Trt)-Gly-Pro-Gln(Trt)) from step 5 was carried out using 70 mL of TFA/TIS/H_2_O (TIS: Triisopropylsilane) in 90/5/5 ratio and stirred for 2.5 hours. The reaction mixture was transferred to a three-neck 1 L round bottom flask containing 700 mL of *tert*-butyl methyl ether at 4 ℃. The product was precipitated as a white solid, which was centrifuged and collected, followed by vacuum drying to obtain the crude Cyclo(Gly-Pro-Gln-Gly-Pro-Gln) peptide as a white powder (Scheme 6). The crude product was then purified by reverse phase C18 preparative chromatography followed by lyophilization to get highly pure Cyclo(Gly-Pro-Gln-Gly-Pro-Gln) peptide (**6**) (Purity 99.2%). ^1^HNMR data is shown in **Table SI**, whereas ^13^CNMR data is present in **Table SII**. HRMS (ESI-TOF) *m/z*: [M + Na]^+^ calcd for C_24_H_36_O_8_N_8_Na, 587.2554; found, 587.2549

**Table S1**. ^1^H-NMR Data (D_2_O, 600 MHz) of Compound **CHP-9** (*δ* in ppm and *J* in Hz)

| No. | Chemical shift (δ in ppm) | Number of  Proteins | ^1^H-^1^H COSY |
| --- | --- | --- | --- |
| 3 | 3.32-3.39,3.79-4.19 | 2 | / |
| 6 | 4.10-4.11,4.25-4.26,4.38-4.38 |  | H22 |
| 9 | 4.25-4.26,4.36-4.38,4.55-4.58 | 1 | H25 |
| 12 | 3.32-3.39,3.79-4.19 | 2 | / |
| 15 | 4.10-4.11,4.25-4.26,4.38-4.38 | 1 | H28 |
| 18 | 4.25-4.26,4.36-4.38,4.55-4.58 | 1 | H19 |
| 19 | 1.86-1.94,2.14-2.31 | 2 | H18, h20 |
| 20 | 1.76-1.86,1.96-2.06 | 2 | H19, H21 |
| 21 | 3.41-3.53,3.59-3.69 | 2 | H20 |
| 22 | 1.87-2.05,2.21-2.25 | 2 | H6, H23 |
| 23 | 2.07-2.41 | 2 | H22 |
| 25 | 1.86-1.94,2.14-2.31 | 2 | H9, H26 |
| 26 | 1.76-1.86,1.96-2.06 | 2 | H25, H27 |
| 27 | 3.41-3.53,3.59-3.69 | 2 | H26 |
| 28 | 1.87-2.05,2.21-2.25 | 2 | H15.H29 |
| 29 | 2.07-2.41 | 2 | H28 |

**Table SII**. ^13^C-NMR Data (D_2_O, 600 MHz) of Compound **CHP-9** (*δ* in ppm and *J* in Hz)

| Entry | Chemical shift (δ in ppm) | DEPT | HSQC  δ(ppm) | HMBC  (Correlated proton signals) |
| --- | --- | --- | --- | --- |
| 2 | 172.18-173.26 | *Quat.* | / | H3, H21 |
| 3 | 42.21 | *Sec.* | 3.32-3.39,3.79-4.19 | / |
| 5 | 175.62 | *Quat.* | / | H3, H6, H22 |
| 6 | 62.44 | *Tert.* | 4.10-4.11,4.25-4.26,4.38-4.38 | H22, H23 |
| 8 | 174.71 | *Quat.* | / | H6, H9, H25 |
| 9 | 53.78 | *Tert.* | 4.25-4.26,4.36-4.38,4.55-4.58 | H25, H26, H27 |
| 11 | 174.09 | *Quat.* | / | H9, H12, H27 |
| 12 | 41.59 | *Sec.* | 3.32-3.39,3.79-4.19 | / |
| 14 | 174.17 | *Quat.* | / | H12, H15, H28 |
| 15 | 60.96-61.59 | *Tert.* | 4.10-4.11,4.25-4.26,4.38-4.38 | H28, H29 |
| 17 | 168.76-169.79 | *Quat.* | / | H15, H18, H19 |
| 18 | 52.26-52.46 | *Tert.* | 4.25-4.26,4.36-4.38,4.55-4.58 | H19, H20, H21 |
| 19 | 26.72 | *Sec.* | 1.86-1.94,2.14-2.31 | H18, H20, H21 |
| 20 | 24.53-24.69 | *Sec.* | 1.76-1.86,1.96-2.06 | H18, H19,H21 |
| 21 | 47.40 | *Sec.* | 3.41-3.53,3.59-3.69 | H18, H19, H20 |
| 22 | 29.26 | *Sec.* | 1.87-2.05,2.21-2.25 | H6, H23 |
| 23 | 31.28-31.57 | *Sec.* | 2.07-2.41 | H6, H22 |
| 24 | 178.00-178.003 | *Quat.* | / | H22, H23 |
| 25 | 26.43 | *Sec.* | 1.86-1.94,2.14-2.31 | H9, H26, H27 |
| 26 | 22.02 | *Sec.* | 1.76-1.86,1.96-2.06 | H9, H25, H27 |
| 27 | 46.69-46.73 | *Sec.* | 3.41-3.53,3.59-3.69 | H9, H25, H26 |
| 28 | 29.21 | *Sec.* | 1.87-2.05,2.21-2.25 | H15, H29 |
| 29 | 31.05-31.16 | *Sec.* | 2.07-2.41 | H15, H28 |
| 30 | 177.71 | *Quat.* | / | H28, H29 |

**^1^H NMR spectra of compound** **CHP-9 and detailed views**


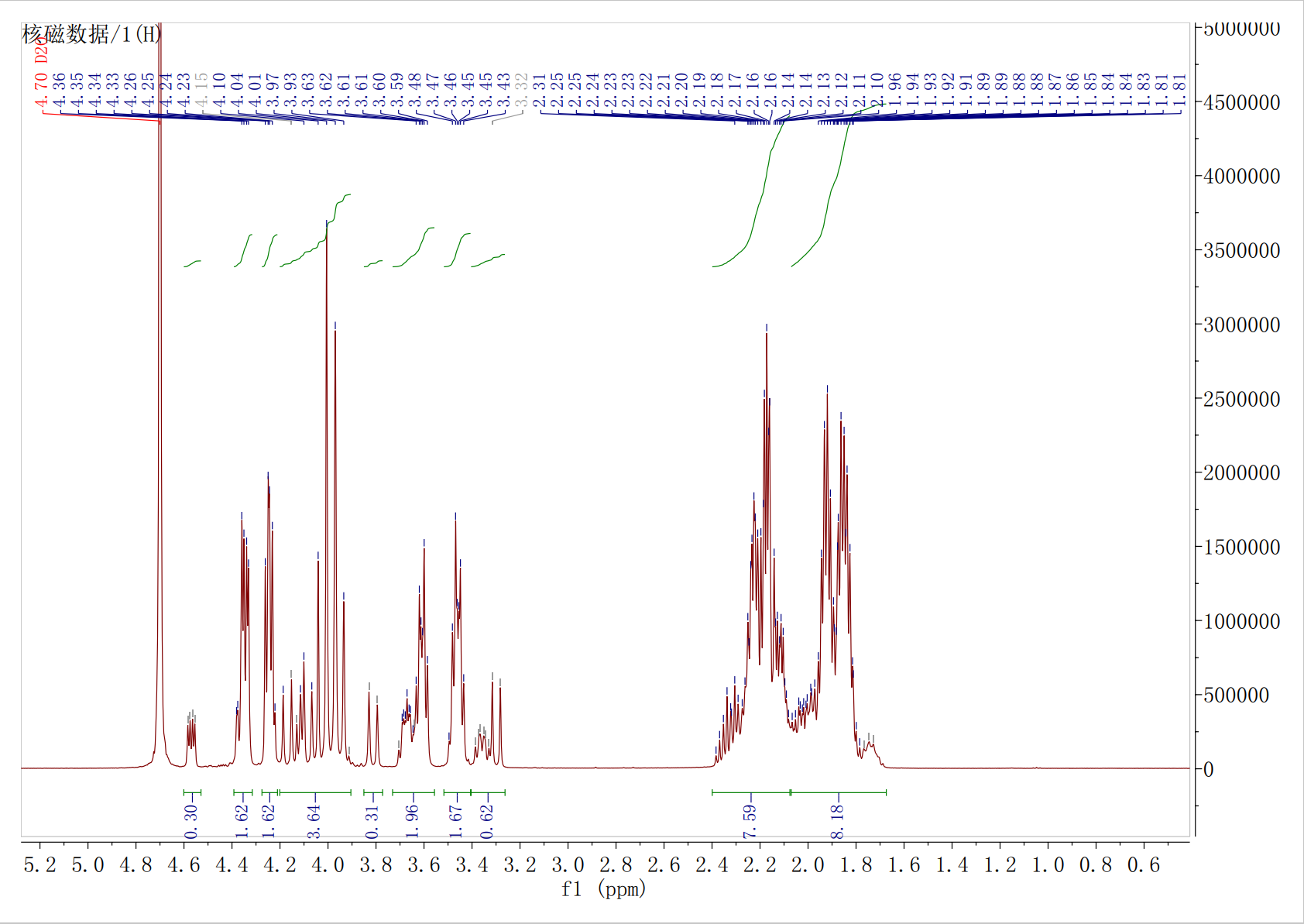


Full view of ^1^H NMR spectrum of compound CHP-9


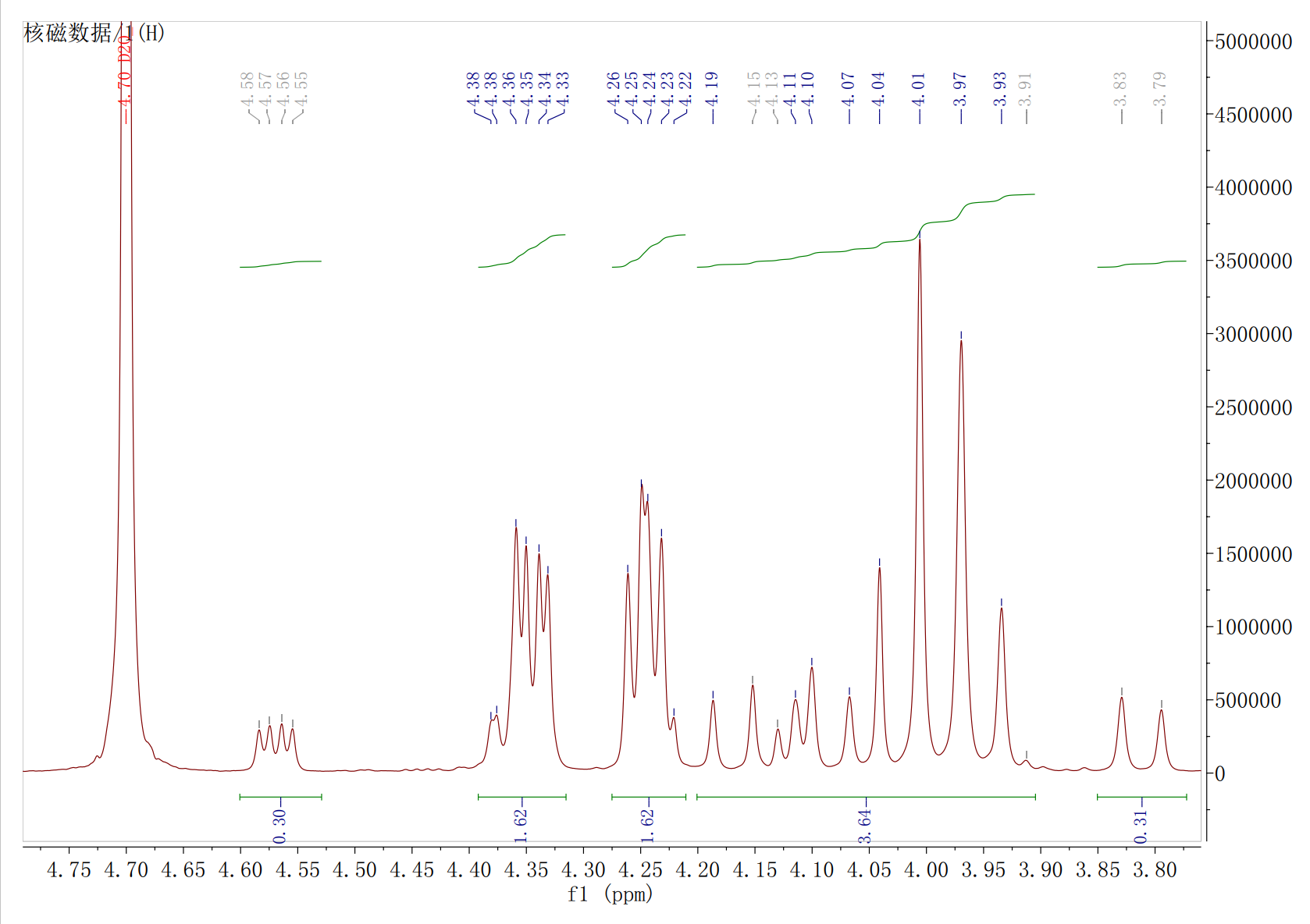


Detailed view (δ: 3.80-4.75 ppm) of ^1^H NMR spectrum of compound CHP-9


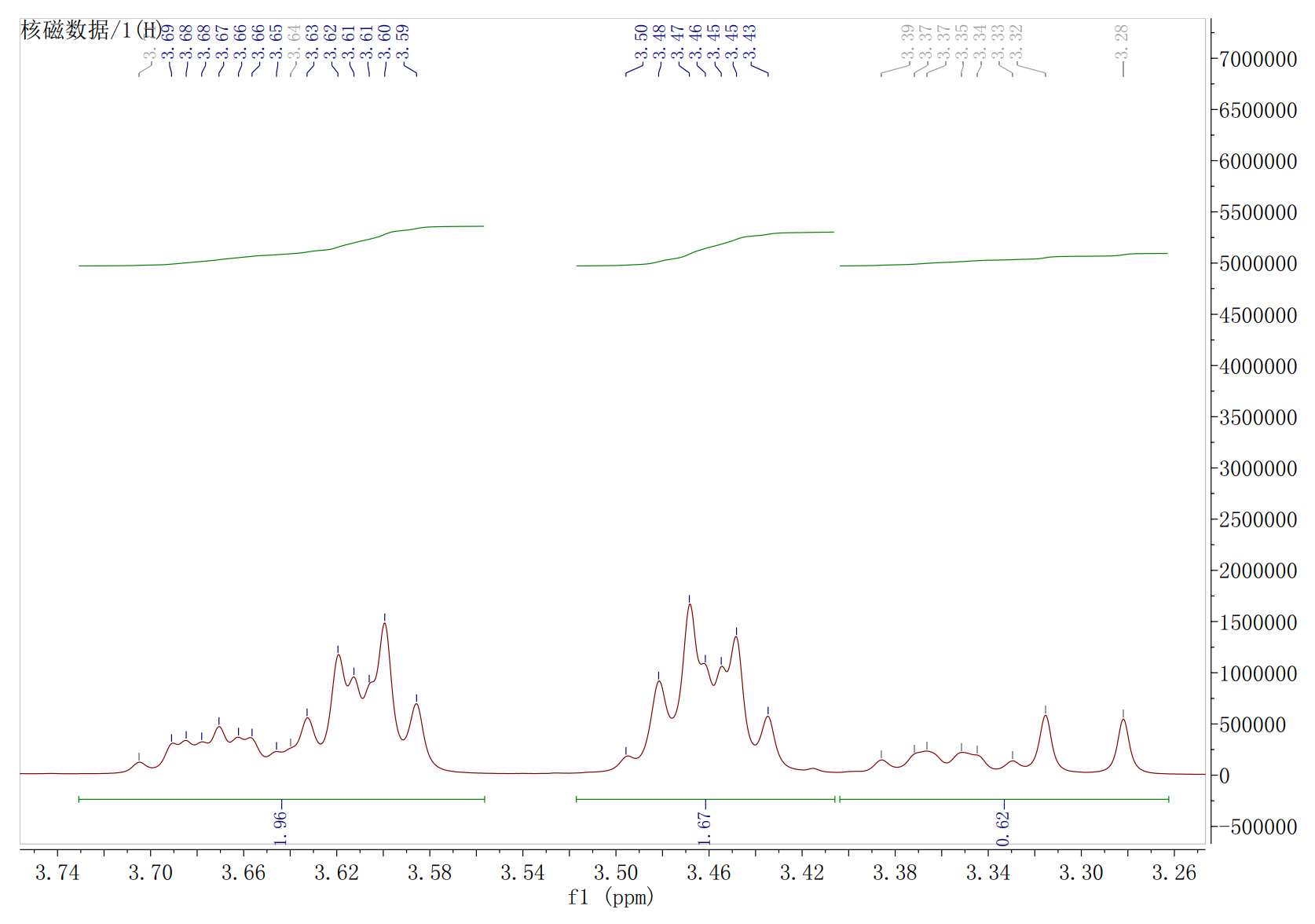


Detailed view (δ: 3.26-3.74 ppm) of ^1^H NMR spectrum of compound CHP-9


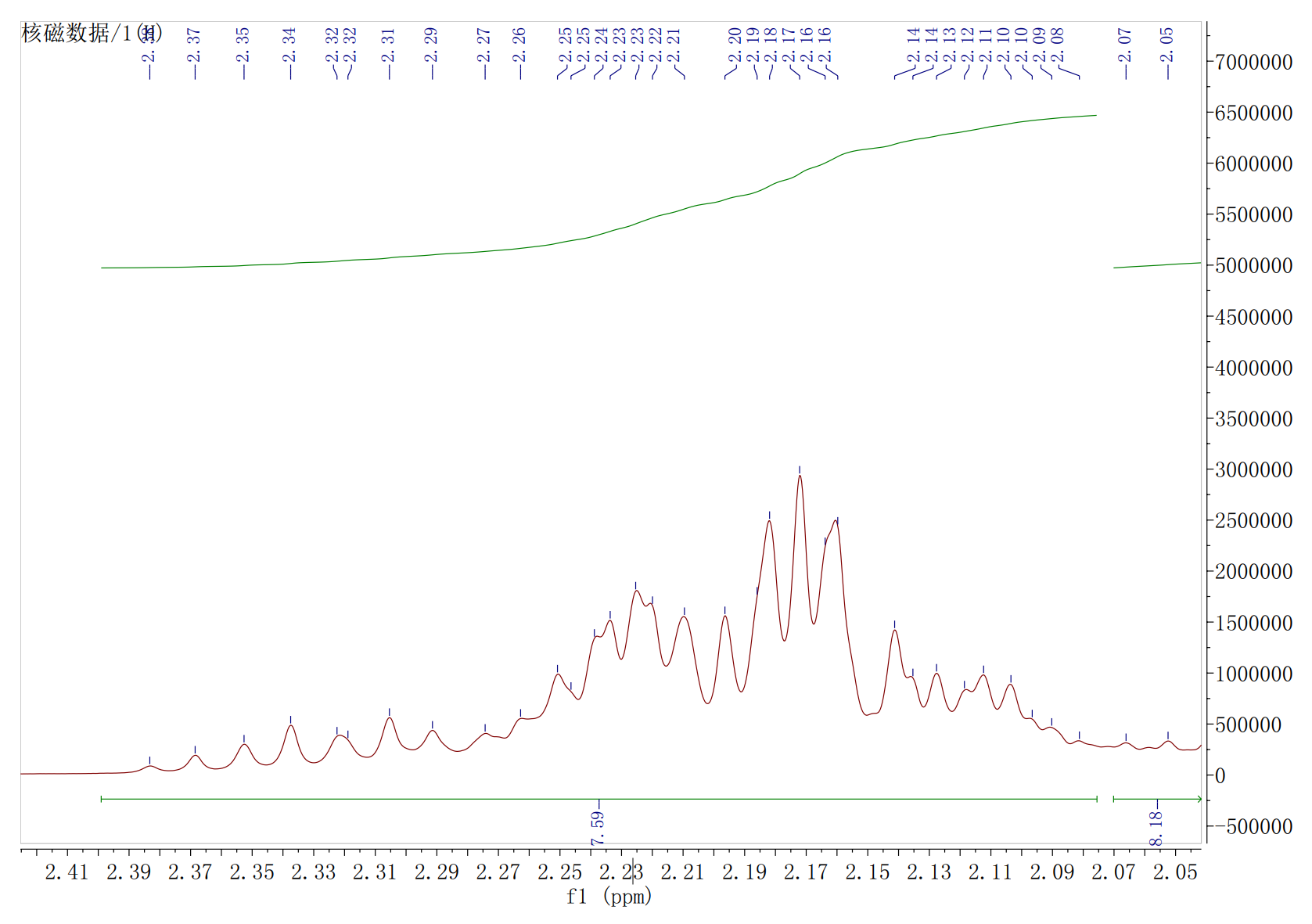


Detailed view (δ: 2.05-2.41 ppm) of ^1^H NMR spectrum of compound CHP-9


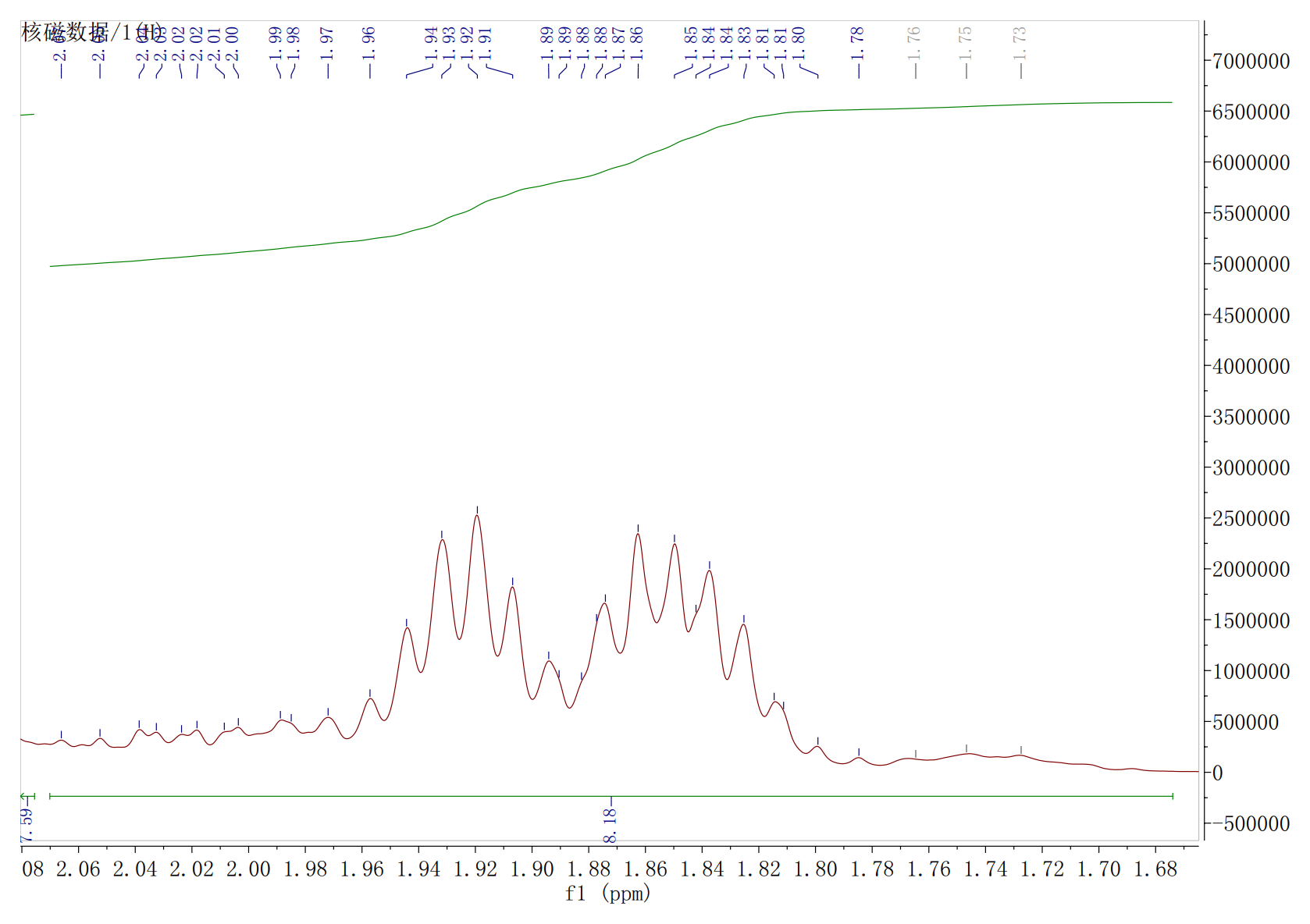


Detailed view (δ: 1.68-2.06 ppm) of ^1^H NMR spectrum of compound CHP-9

^13^C NMR spectra of compound CHP-9 and assignment of signals.

DETP90


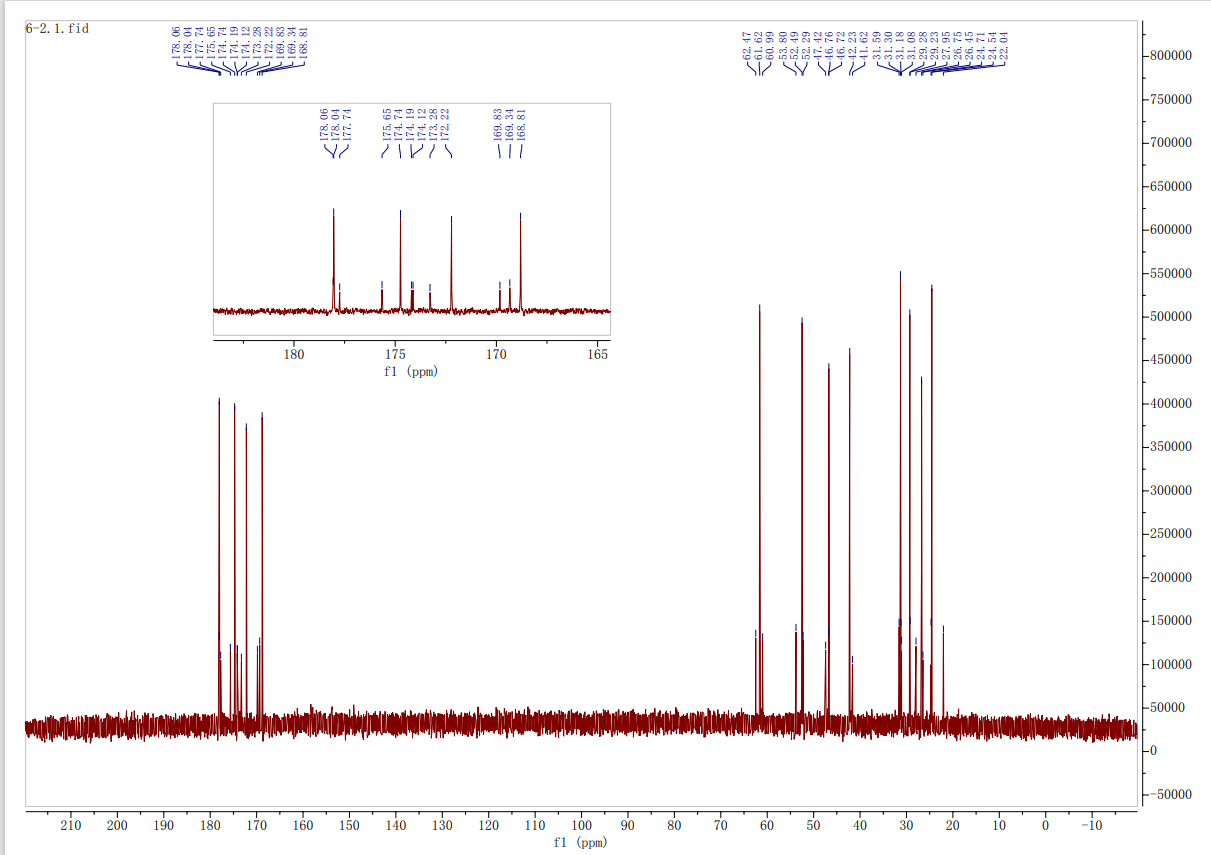


DEPT135


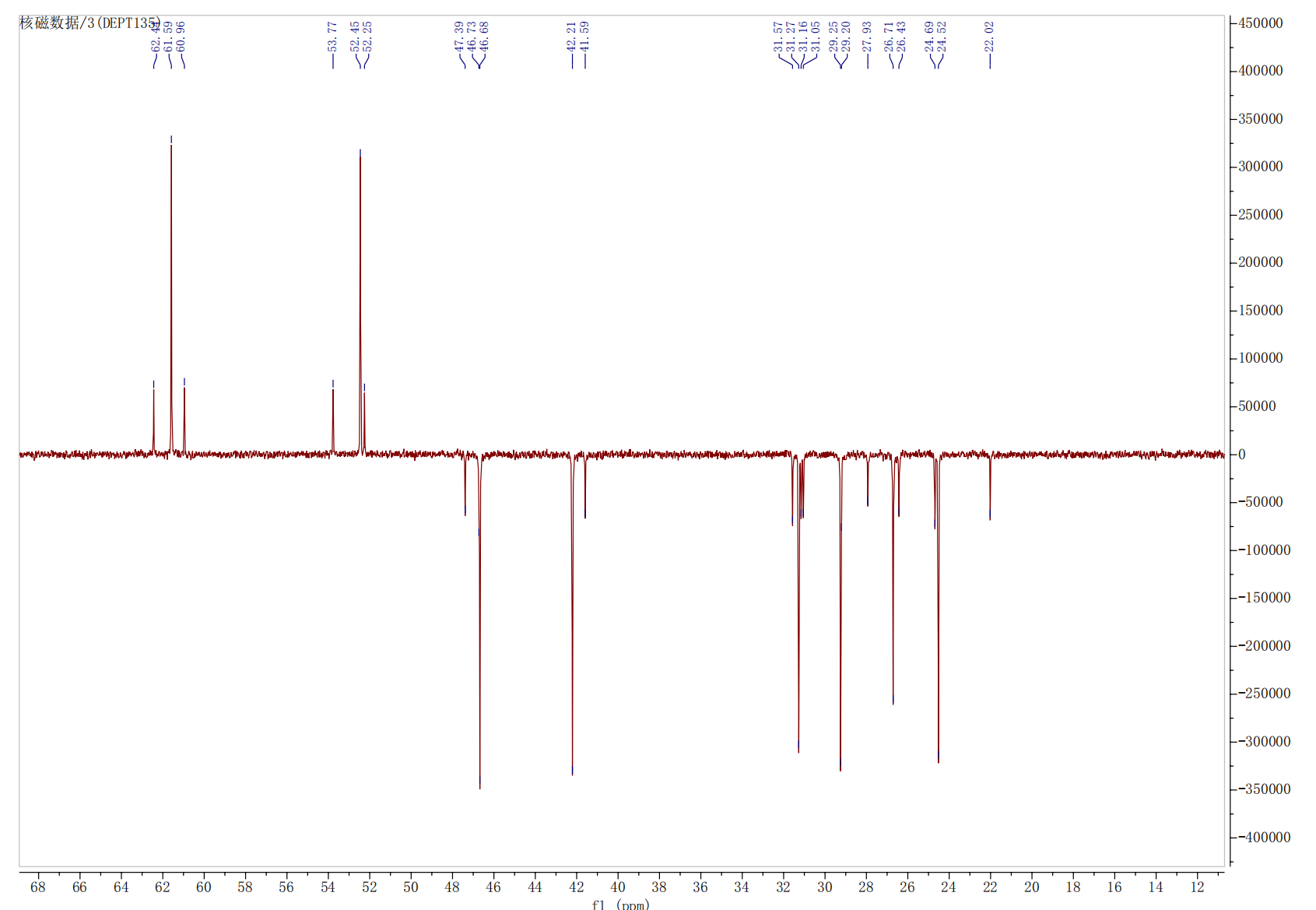


**Mass spectrum of compound CHP-9**

HRMS spectra of Compound **CHP-9** (ESI, positive mode)

**Reference:**

[1] **Liu S, Yang W, Gao Y.** Cyclopeptide CHP-9 for skin lightening applications. **Chinese Patent CN 116284256 A.** Published **2023** Jun 23.
